# The Influence of a Juvenile’s Abuse History on Support for Sex Offender Registration

**DOI:** 10.1037/law0000028

**Published:** 2014-11-17

**Authors:** Margaret C. Stevenson, Cynthia J. Najdowski, Jessica M. Salerno, Tisha R. A. Wiley, Bette L. Bottoms, Katlyn S. Farnum

**Affiliations:** 1 Department of Psychology, The University of Evansville; 2 Department of Criminal Justice, University at Albany, State University of New York; 3 Department of Social and Behavioral Sciences, Arizona State University; 4 National Institute on Drug Abuse, Rockville, Maryland; 5 Department of Psychology, The University of Illinois at Chicago; 6 Department of Psychology, The University of Nebraska-Lincoln

**Keywords:** child sexual abuse, juvenile sex offending, legal decision making, attributions, public policy

## Abstract

We investigated whether and how a juvenile’s history of experiencing sexual abuse affects public perceptions of juvenile sex offenders in a series of 5 studies. When asked about juvenile sex offenders in an abstract manner (Studies 1 and 2), the more participants (community members and undergraduates) believed that a history of being sexually abused as a child causes later sexually abusive behavior, the less likely they were to support sex offender registration for juveniles. Yet when participants considered specific sexual offenses, a juvenile’s history of sexual abuse was not considered to be a mitigating factor. This was true when participants considered a severe sexual offense (forced rape; Study 3 and Study 4) and a case involving less severe sexual offenses (i.e., statutory rape), when a juvenile’s history of sexual abuse backfired and was used as an aggravating factor, increasing support for registering the offender (Study 3 and Study 5). Theoretical and practical implications of these results are discussed.

Concerns about protecting society from dangerous sex offenders have not only provided justification for adult sex offender registry laws, but also for extending the registry to juveniles (Caldwell, Ziemke, & Vitacco, 2008; Salerno, Stevenson, et al., 2010; SORNA; 42 U.S.C. § 16911), who differ in important ways from adult offenders (for reviews, see Chaffin, 2008; Trivits & Reppucci, 2002). These laws are presumed to protect society, but there is little evidence that they are effective at decreasing sex offender recidivism (Adkins, Huff, & Stageberg, 2000; Caldwell & Dickinson, 2009; Letourneau & Armstrong, 2008; Sandler, Freeman, & Socia, 2008; Schram & Milloy, 1995). They might even harm offenders (Levenson & Cotter, 2005; Levenson, D’Amora, & Hern, 2007; Tewksbury, 2005; Tewksbury & Lees, 2006, 2007) in ways that psychologists fear might increase their likelihood of committing future sex offenses (Letourneau & Miner, 2005; Freeman & Sandler, 2010; Trivits & Reppucci, 2002).

It is possible that the recent application of registry laws to juvenile sex offenders might be motivated by politicians’ assumption that the public endorses such policies (e.g., Meloy, Boatwright, & Curtis, 2013; Sample & Kadleck, 2008). Recent research has tested this assumption. Kernsmith, Craun, and Foster (2009) found that 86% of respondents agreed that a juvenile under age 18 who forces someone to have sex should be required to register as a sex offender. Even so, the juvenile was perceived as less worthy of registration than someone who sexually abused a child. The authors were not explicit about the offender’s age in the comparison cases, however, making it difficult to understand the effect of offender age on registration support. In a series of experimental studies, Salerno, Najdowski, and colleagues (2010) found that the public was highly supportive of registering both juvenile and adult sex offenders, but only when they were asked about support for registry laws in general. When asked about specific cases, participants were significantly less likely to support registration in cases involving (a) younger as compared to older juveniles and (b) less severe offenses, such as sexting, sexual harassment, and statutory rape (offenses for which juveniles are registered in some states), as compared to more forced and violent forms of rape. Indeed, support for juvenile sex offender registration is influenced by a variety of factors, including the education level of the individual (Stevenson, Smith, Sekely, & Farnum, 2013) and the race (Stevenson, Sorenson, Smith, Sekely, & Dzwairo, 2009) and sexual orientation (Salerno, Murphy, & Bottoms, 2014) of the offender and victim.

Although neither adult (Hanson & Bussiere, 1998; Widom & Ames, 1994) nor juvenile sex offenders (Rasmussen, 1999; Silovsky & Niec, 2002) are especially likely to have a history of experiencing child sexual abuse themselves, many laypersons, legislators, and other legal decision makers believe that a majority of adult sex offenders were abused themselves (Fortney, Levenson, Brannon, & Baker, 2007; Levenson, Brannon, Fortney, & Baker, 2007; Sample & Kadleck, 2008). This inaccurate belief might also extend to juvenile sex offenders, and in turn, influence legal action taken against them, a possibility that motivated the present research. In five studies, we investigated (a) the extent to which laypeople believe that juveniles commit sex offenses because they were themselves sexually abused as children (Study 1), (b) how such beliefs influence laypeople’s support for juvenile sex offender registration in general (Study 1 and Study 2), (c) whether such beliefs influence support for registering juveniles differently depending on the specific type of sex offense committed (Study 3), and (d) how the experimental manipulation of a juvenile sex offender’s childhood abuse history influences support for sex offender registration for a more severe offense (forced rape, Study 4) and for a less severe offense (statutory rape, Study 5). Before describing the experiments, we review the extant literature regarding laypersons’ beliefs about the prevalence of childhood sexual abuse among juvenile sex offenders and how it influences perceptions of them. With attribution theory as a guiding framework, we make predictions about the effects of prior beliefs on registration support and on the underlying psychological processes.

## Beliefs About and Effects of Offenders’ Experiences of Childhood Sexual Abuse

Hanson and Slater’s (1988) work suggests that the child sexual abuse rate for adult sex offenders is 28%, while Ryan, Miyoshi, Metzner, Krugman, and Fryer (1996; for review, see Worling, 2001) estimates it to be around 31–39% for juvenile sex offenders. Yet the public believes as many as 67% of adult offenders were sexually abused themselves (Fortney et al., 2007; Levenson, Brannon, Fortney, & Baker, 2007). We predicted that the public would overestimate the prevalence of sexual abuse histories among juvenile sex offenders as they do for adult sex offenders. Further, we investigated the prevalence of this belief and its link to support for registry laws, given that Fortney et al. (2007) and Levenson et al. (2007) argue that people assume early sexual abuse leads to future sexual offending.

Some studies have revealed that an adult defendant’s history of child abuse leads perceivers to have a more positive reaction to those defendants (Garvey, 1998; Heath, Stone, Darley, & Grannemann, 2003; Lynch & Haney, 2000). For example, Stalans and Henry (1994) asked community members to read a vignette describing a 16-year-old boy accused of killing either his father or a neighbor following a heated argument. Participants were less likely to think that the boy intended to kill the victim or understood the wrongfulness of his actions when he was portrayed as having been abused by his father than when not. Those inferences decreased participants’ recommendations that the abused boy be transferred from juvenile court to adult criminal court. Using the same experimental design, Nunez, Dahl, Tang, and Jensen (2007) found that mock jurors were less likely to endorse retributive goals (i.e., a “just desserts” desire to get even with the juvenile) for punishing an abused versus a nonabused juvenile offender, and in turn, less likely to recommend that the abused juvenile be transferred from juvenile court to adult criminal court.

Similarly, Najdowski, Bottoms, and Vargas (2009) found that mock jurors believed that a girl’s history of neglect, physical abuse, and sexual abuse might have contributed to various offenses (shoplifting, drug offense, murder in self-defense, aggravated murder). They perceived an abused (vs. nonabused) offender as less deviant, less responsible, and more amenable to rehabilitation—but only when the crime was murder in self-defense against the perpetrator of abuse. In contrast, jurors perceived an abused girl as less amenable to rehabilitation than a nonabused girl when she had committed aggravated murder. Thus, even though jurors intuit the link between childhood abuse and later criminal behavior (i.e., “the cycle of violence,” Widom, 1989), they may not account for past abuse when determining a juvenile’s criminal culpability and may even use it as evidence of future dangerousness, especially when the juvenile commits a crime against someone other than the perpetrator of abuse.

Thus, the evidence is mixed regarding whether offenders are perceived more favorably if they have been abused than if not. Grisso (2002) even expressed concern that expert witnesses might link a juvenile’s history of being abused with being permanently damaged and less amenable to rehabilitation. Indeed, there is evidence that physically abused juveniles are treated more severely within the juvenile justice system, likely because they are perceived as “lost causes” (Stevenson, 2009). This is not consistent with laws mandating that a juvenile’s abuse history should be used as a mitigating factor when deciding whether to transfer a juvenile to adult court (e.g., Illinois’ Juvenile Court Act, 1987). Moreover, defense attorneys might hope or even assume that a juvenile’s history of being sexually abused as a child will be used as a mitigating factor and reduce sentence severity—an assumption that has yet to be empirically tested. The goal of the present research is to explore assumptions and myths people have about juvenile sex offenders, and to test how those influence their support for registration policies.

## Attribution Theory

Attribution theory is useful for disentangling these mixed findings. Attributions, or inferences about the causes of behavior, are made with regard to three aspects of behavior: (a) *locus*, or whether the causal factor is within the actor (i.e., internal) versus the environment (i.e., external); (b) *stability*, or whether the causal factor is constant (i.e., stable) versus changing (i.e., unstable) over time; and (c) *controllability*, or whether the actor does (i.e., controllable) or does not (i.e., uncontrollable) possess the ability to change his or her behavior (for reviews, see Shaver, 1985; Weiner, 2006). According to attribution theory (Weiner, 2006), different attributions produce specific, reliable predictions about perceivers’ (a) judgments regarding a transgressor’s responsibility for the crime (i.e., not responsible vs. responsible), (b) affective reactions to the case (i.e., sympathy vs. anger), and (c) sentencing goals (i.e., utilitarian vs. retributive). Jurors should render more punitive case judgments when the cause of a transgression is perceived to have been internal, controllable, and stable, as opposed to external, uncontrollable, and unstable (Shaver, 1985; Weiner, 2006). In fact, research has shown that the less people perceive the cause of a crime to be internal, controllable, or stable, the less punitively they treat the offender (Carroll, 1978; Carroll & Payne, 1977; Graham, Weiner, & Zucker, 1997; Najdowski & Bottoms, 2012).

Graham and colleagues (1997), however, theorized that different attributions could influence different punishment goals and, in turn, punishment severity. Specifically, perceiving that an offender had control of his or her criminal behavior would increase retributive goals for punishing the offender (i.e., the defendant should “get what he deserves”). In contrast, perceiving that the cause of the offender’s criminal behavior is stable would increase one’s utilitarian goals for punishing the offender (i.e., concern for protecting society). Either of these attributional processes is expected to increase punitiveness toward an offender. It is this model that is particularly useful for understanding reactions to previously abused juvenile sex offenders.

## Why a Juvenile Sex Offender’s Abuse History Could Be a Mitigating or Aggravating Factor

On the one hand, believing that juveniles commit sex offenses because they were abused themselves as children might be associated with more uncontrollable attributions and diminished retributive goals, consistent with prior research (e.g., Graham et al., 1997; Najdowski et al., 2009). In turn, these attributions and goals might be associated with less support for the application of sex offender registry laws to juveniles. In support, Stevenson, Bottoms, and Diamond (2010) found that many mock jurors made uncontrollable attributions for an adult defendant who had a history of being physically abused as a child by arguing that the abuse explained his inability to control his adult criminal behavior (i.e., murder). The more jurors endorsed such uncontrollable attributions, the more they supported a sentence of life versus death. In addition, retributive goals play a major role in explaining public support for registry laws: People are less likely to support registry laws for juveniles who commit less versus more severe sex offenses (Salerno, Najdowski et al., 2010), who perpetrate offenses against victims of the same race versus different race (Stevenson, Najdowski, Bottoms, & Haegerich, 2009), and who perpetrate male-on-female versus male-on-male offenses (Salerno et al., 2014) because they are less motivated to punish such juveniles. Thus, believing that juveniles perpetrate sex offenses because they were sexually abused themselves could have a mitigating effect on registration support.

On the other hand, believing juveniles commit sex offenses because they were sexually abused might lead to more stable attributions that juvenile sex offenders are permanently damaged and likely to reoffend, as well as internal attributions that juveniles commit sex offenses because of mental illness or sexual deviance. Such beliefs might increase utilitarian motives to protect society and, in turn, support for the registry. In support, Stevenson and colleagues (2010) illustrated that the more likely jurors were to argue that physical abuse causes permanent psychological damage (a stable, internal attribution), the more likely they were to recommend a sentence of death versus life for an adult defendant who had been physically abused as a child. Also, Sample and Kadleck’s (2008) survey revealed that legislators and other decision makers perceive sex offenders as incurable and destined to reoffend over and over again, and cite this belief as justification for sex offender legislation. In addition, Salerno, Stevenson, and colleagues (2010) found that people overestimated the likelihood that juvenile sex offenders would commit future offenses (although estimates were lower for juvenile than adult sex offenders). According to attribution theory, such stable attributions lead to increased utilitarian goals to protect society and, in turn, sentence severity (Graham et al., 1997; Weiner, 2006). In fact, utilitarian goals to protect society explain why the public supports more punitive sex offender registry laws for offenders who are older or commit more severe offenses compared to those who are younger or commit less severe offenses (Salerno, Najdowski et al., 2010). Thus, beliefs about the effects of prior abuse on later sexual offending could have an aggravating effect on registration support.

## Study 1

In Study 1, we investigated people’s estimates of the prevalence of sexual abuse among juvenile sex offenders, and whether they believe such abuse explains why juveniles commit sex offenses. We also tested our competing hypotheses (discussed above) to determine whether such beliefs would relate to decreased or increased support for applying registry laws to juvenile sex offenders.

### Method

#### Participants

Participants were 127 community members from a Midwestern urban area: 79% women, 37 years old on average (*SD* = 13, ranging from 18 to 70 years), and predominantly Caucasian (81%, with 5% Asian, 4% African American, 4% Hispanic, and 7% of other ethnicities).

#### Materials

An online survey first provided participants with the following information and instructions (developed by Salerno, Najdowski et al., 2010):

Adults found guilty of a sex offense must be listed on a public sex offender registry. In various states, this registry includes information such as name, social security number, age, race, gender, birth date, physical description, address, place of employment, details about the offense(s), fingerprints, a photo, a blood sample, and a hair sample. This information is available to the public upon request, sometimes by being posted on the Internet. In some cases, the police directly notify the people who live in the same area as the registered sex offender. Sex offenders are required to register anywhere from a few years to their entire life, depending on the state.

We are interested in your thoughts about applying these registration laws to juveniles (16 years old or younger) who have been adjudicated (found guilty in juvenile court) or convicted as sex offenders.

The survey then assessed participants’ beliefs about (a) the prevalence of sexual abuse among juvenile sex offenders, (b) the link between sexual abuse and later offending, and (c) support for applying registry laws to juvenile sex offenders.

##### Spontaneous attributions of sex offending to sexual abuse

Because we were interested in participants’ spontaneous attributions about the causes of sex offending among juveniles, we first asked, “Why do you think the typical juvenile sex offender commits his or her crimes?” This question appeared alone on the computer screen before all other questions to ensure that responses were not biased by any subsequent measures. Modeled after coding methods in Stevenson et al. (2010), responses were coded for references to (a) sexual abuse (e.g., “They were previously sexually abused”); (b) abuse of an unspecified nature (e.g., “Maybe they were abused when they were young”); (c) some other dysfunctional background (e.g., “Lack of home support and or guidance”); or (d) none of the above (e.g., “Mental issues”). Two independent raters coded 50% of responses and were reliable on each code (proportion of agreement ≥99%). Disagreements were resolved by discussion and one rater coded the remaining data. Four participants (3%) did not respond to this item.

##### Registration support

Next, participants were asked to agree or disagree with the following statement, our measure of registration support: “Public registration laws are too severe for juvenile sex offenders,” using a scale ranging from 1 (*strongly disagree*) to 5 (*strongly agree*). This was reverse-scored so that higher scores reflect greater support for the registry.

##### Direct attributions of sex offending to sexual abuse

On a separate computer screen, participants were asked to respond to the statement, “Many juvenile sex offenders commit sex offenses because they were sexually abused themselves,” using a 5-point scale ranging from 1 (*strongly disagree*) to 5 (*strongly agree*). This item was modeled after a similar item used by Bumby and Maddox (1999).

##### Estimates of abuse prevalence

Participants then provided estimates of sexual abuse prevalence by responding on an 11-point scale (ranging from 0% to 100% in intervals of 10%) to the question, “In your opinion, what percentage of all juvenile sex offenders were sexually abused as children?” (modeled after an item used by Levenson et al., 2007).

#### Demographics

Finally, participants were asked to provide their gender, age, and ethnicity.

#### Procedure

Community members were recruited via postings on http://www.craigslist.org in various U.S. cities. They (a) were informed that their participation was voluntary and anonymous, (b) received all instructions and completed all measures online using SurveyMonkey.com web survey software, and (c) were thanked and debriefed, in keeping with an approved Institutional Review Board (IRB) protocol.

### Results

Most participants (62%) spontaneously attributed sexual offending to previous abuse or other dysfunctional background factors: 31% (*n* = 38) cited prior sexual abuse as a cause, 9% (*n* = 11) mentioned abuse of an unspecified nature, and 22% (*n* = 27) mentioned other dysfunctional background factors. When subsequently asked directly, 84% of participants agreed or strongly agreed that “Many juvenile sex offenders commit sex offenses because they were sexually abused themselves” (*M* = 4.12, *SD* = .74).

On average, participants estimated that 65% (*SD* = 23%, Range = 10–100%) of juvenile sex offenders have been sexually abused themselves. A linear regression analysis using the direct measure of attributions of sexual offending to sexual abuse revealed that the more participants agreed that juveniles commit sex offenses because they were abused themselves, the less they supported the registry for juveniles, β = −.28, *t*(112) = −3.11, *p* < .01, *R* = −.28, *R*^2^ = .08, *F*(1, 112) = 9.65, *p* < .01.

### Discussion

As predicted, when asked about juvenile sex offenders in general, many people believe that juvenile sex offenders have had prior experiences of abuse or other dysfunctional backgrounds—both when asked to report their spontaneous thoughts about why juveniles commit sex offenses and when asked directly about sexual abuse. These estimates are nearly exactly the same as Levenson et al. (2007) found for adult sex offenders (67%) and more than double the actual prevalence among boy sex offenders (31%, Worling, 2001). Further, the more participants believed that sexual abuse causes sexual offending, the less they supported registry laws for juvenile sex offenders. This is consistent with other findings that abuse is sometimes used as a mitigating factor (e.g., Najdowski et al., 2009; Nunez et al., 2007; Stalans & Henry, 1994), and demonstrates that this effect extends to public support for juvenile sex offender registration policies.

## Study 2

Study 2 was designed to replicate and extend the results of Study 1 with a larger and more diverse sample and to explore whether attributions about why juveniles commit sex offenses explain the mitigating effect of beliefs about abuse on registration support found in Study 1. We hypothesized that greater agreement that sexual abuse causes sex offending would be associated with increased uncontrollable attributions and decreased retributive punishment goals, which would, in turn, decrease registration support.

Although Study 1 showed that attributing sex offending to past abuse is associated with diminished registration support, we still tested for the competing possibility that this belief would increase registration support due to stable and internal attributions, because it is still possible that people who attribute sex crimes to past abuse also believe that juvenile sex offenders who experienced sexual abuse have deviant sexual arousal and mental illness (internal attributions), which cause them to commit sex crimes. Such beliefs might increase expectations that a juvenile sex offender will commit future sex crimes (stable attributions). Even so, given the results of Study 1, we did not anticipate that these beliefs would translate into greater registration support. As theorized by Weiner (2006), internal attributions (i.e., mental illness) and stable attributions (i.e., likely recidivism) do not always translate into unfavorable judgments when behavioral causes are also perceived to be the result of uncontrollable factors—in this case, a prior history of sexual abuse. Thus, we expected that the mitigating influence of uncontrollable attributions and diminished retributive goals would override the hypothesized aggravating effects of internal and stable attributions that stem from believing sexual abuse contributes to sexual offending.

### Method

#### Participants

Participants were a diverse, urban group of undergraduates at a large Midwestern research university (*n* = 87), and community members (*n* = 91) who were 18 years old or older. Undergraduates were 66% women, 19 years old on average (*SD* = 2, ranging from 18 to 30 years), and 41% Caucasian, 32% Asian, 12% African American, 12% Hispanic, and 4% of other ethnicities. Community members were 55% women, 37 years old on average (*SD* = 13, ranging from 18 to 80 years), and 57% Caucasian, 10% African American, 15% Asian, 14% Hispanic, and 3% of other ethnicities. The two samples were combined for analyses because, importantly, preliminary analyses revealed no significant differences in their responses, all βs ≤ .13, all *ns*.

#### Materials

A questionnaire included the same measures used in Study 1 in addition to items assessing uncontrollable, internal, and stable attributions for juveniles’ sex offending behavior and participants’ retributive and utilitarian punishment goals. Unless otherwise noted, all responses were made on 6-point scales ranging from −3 (*strongly disagree*) to + 3 (*strongly agree*) with no midpoint. Values were transformed to create scales ranging from 1 (*strongly disagree*) to 6 (*strongly agree*).

To measure uncontrollable attributions, participants indicated their agreement with the statement: “Juvenile sex offenders are unable to control their behavior.”

To assess internal attributions of juveniles’ sex offenses, participants indicated agreement with the following statements: “Many juvenile sex offenders commit sex offenses because of deviant sexual arousal,” “In your opinion, what percentage of all juvenile sex offenders are severely mentally ill?” Responses were given on an 11-point scale ranging from 0% to 100% in intervals of 10%. These measures were modeled after items used in prior research examining perceptions of juvenile offenders in general (Haegerich & Bottoms, 2004; Najdowski & Bottoms, 2012; Najdowski et al., 2009; Vidal & Skeem, 2007) and sex offenders specifically (Bumby & Maddox, 1999; Malesky & Keim, 2001; Proeve & Howells, 2006).

To measure stable attributions, participants were asked, “In your opinion, what percentage of all juvenile sex offenders eventually commit another sex offense?” Responses were made on the 11-point percentage scale. To measure retributive goals, participants indicated how much they agreed that, “I would support the sex offender registry for juveniles, even if there is no scientific evidence showing that it reduces sexual abuse.” To measure utilitarian goals, participants indicated how much they agreed that, “Juvenile sex offenders pose a danger to society.”

#### Procedure

Undergraduates completed the questionnaire with items in the order described above during a mass-testing session, along with various unrelated surveys. Community members completed the questionnaire after being approached in various public settings in a large metropolitan area (mainly trains, but also airports, malls, etc.). All participants were told that their participation was voluntary and anonymous, then thanked for their participation, in keeping with IRB-approved procedures. Undergraduates were compensated with course credit for participating and community members received no compensation.

### Results

As in Study 1, many participants overestimated the proportion of juvenile sex offenders who were sexually abused as children (*M* = 53%, *SD* = 23%, Range = 0–100%) and, when asked directly, 51% of participants agreed or strongly agreed that many juveniles commit sex offenses because they were sexually abused themselves (*M* = 3.50, *SD* = .93).

A series of multiple linear regression analyses tested the effect of beliefs that sexual abuse causes sex offending on (a) uncontrollable, internal, and stable attributions for offending; (b) utilitarian and retributive goals; and (c) support for registry laws.

Greater agreement that abuse contributes to sex offending was associated with (a) significantly less registration support, β = −.25, *t*(173) = −3.32, *p* = .001, *R* = .25, *R*^2^ = .06, *F*(1, 173) = 11.03, *p* < .001; (b) significantly more uncontrollable attributions, β = .17, *t*(173) = 2.33, *p* < .05, *R* = .17, *R*^2^ = .03, *F*(1, 173) = 5.43, *p* < .05; (c) marginally less retributive goals, β = −.15, *t*(172) = −1.92, *p* < .06, *R* = .15, *R*^2^ = .02, *F*(1, 172) = 3.83, *p* = .06; (d) significantly more internal attributions to deviant sexual arousal, β = .18, *t*(173) = 2.46, *p* < .05, *R* = .18, *R*^2^ = .03, *F*(1, 173) = 6.07, *p* < .05, and mental illness, β = .19, *t*(171) = 2.46, *p* < .05, *R* = .18, *R*^2^ = .03, *F*(1, 171) = 6.04, *p* < .05; and (e) marginally greater stable attributions regarding recidivism, β = .13, *t*(169) = 1.70, *p* = .09, *R* = .13, *R*^2^ = .02, *F*(1, 169) = 2.89, *p* = .09. There was no significant relation between agreement that abuse leads to sex offending and utilitarian goals of punishment, β = .01, *t*(174) = .18, *ns*, *R* = .01, *R*^2^ = .00, *F*(1, 174) = .03, *ns*.

**Figure 1 fig1:**
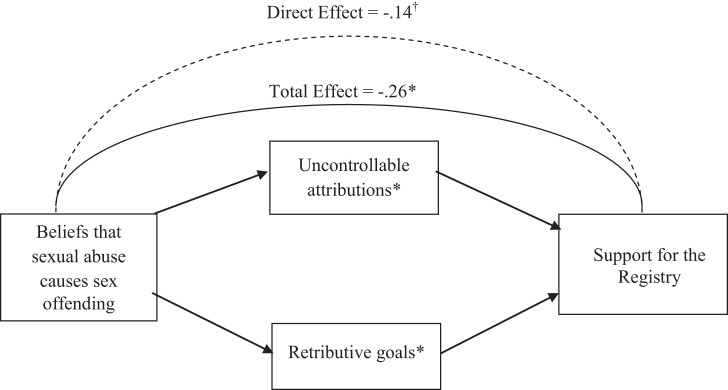
Mediators of the effect of beliefs that sexual abuse causes sex offending on support for the registry (Study 2). ^†^ *p* < .10, * *p* < .05.

Next, we tested for potential mediators of the effect of beliefs that sexual abuse causes sex offending on registration support (see Figure 1). We included as potential mediators only variables that were significantly predicted by abuse attributions in the prior analyses, as recommended by Baron and Kenny (1986). First, we tested whether each potential mediator significantly predicted registration support, finding that neither internal attributions to deviant sexual arousal nor to mental illness emerged as significant predictors, βs < −.10, *t*s < −1.26, *ns*, and thus, they were not included in the mediation model. Although the effect of stable attributions on registration support was significant, β = .23, *t* = 3.11, *p* < .01, this effect was in the opposite direction as the effect of abuse attributions on registration support, instead predicting greater registration support. That is, although greater agreement that abuse contributes to sex offending was associated with less registration support (i.e., a lenient judgment), greater agreement that abuse contributes to sex offending was associated with greater stable attributions (i.e., belief that the juvenile will reoffend)—a variable that predicts greater registration support (i.e., a harsher judgment). Because stable attributions predict greater registration support, and greater abuse attributions predicted greater stable attributions, stable attributions logically cannot explain why abuse attributions predicted reduced registration support. Thus, according to Baron and Kenny (1986), stable attributions logically cannot explain (i.e., mediate) the effect of abuse history on support for the full application of the registry, and so this variable was not considered further. Yet, uncontrollable attributions and retributive goals positively predicted registration support, βs > .23, *t*s > 3.11, *p*s < .01, and were therefore included in the mediation model.[Fig-anchor fig1]

As recommended for research involving multiple mediators, we employed nonparametric bootstrapping analyses (see Preacher & Hayes, 2004; Preacher, Rucker, & Hayes, 2007) to test our meditational model (see Figure 1). According to Preacher and Hayes (2004), for mediation to be significant, the 95% bias-corrected and accelerated confidence intervals for the indirect effects (IE) must not include 0. Results based on 5,000 bootstrapped samples revealed that the total effect (TE) of abuse attributions on registration support was significant (TE = −.26, *SE* = .08, *t* = −2.97, *p* < .05) but the direct effect (DE) was only marginal (DE = −.14, *SE* = .08, *t* = −1.81, *p* = .07). Next, we determined whether the indirect effects of the independent variable on the dependent variable through the three proposed mediators were statistically significant by relying on the following criteria: When zero is not in the 95% confidence interval, the indirect effect is significantly different from zero at *p* < .05 (two-tailed). Both uncontrollable attributions, 95% CI [−.11, −.002] and retributive goals of punishment, 95% CI [−.17, −.01] significantly mediated the relationship between abuse attributions and registration support. Finally, a comparison of the relative strength of the individual indirect effects against each other revealed no significant difference (i.e., the confidence interval included zero), 95% CI [−.06, .14].

### Discussion

Study 2 replicated Study 1’s findings that (a) people overestimate the prevalence of sexual abuse history among juvenile sex offenders and believe that sexual abuse is a precursor to juvenile sex offending, and (b) these beliefs are associated with less support requiring juveniles to register as sex offenders. Study 2 demonstrated that this effect was explained by participants’ uncontrollable attributions and retributive goals, in line with attribution theory (Graham et al., 1997; Weiner, 2006). That is, as predicted, the more participants believed that a history of sexual abuse explains why juvenile sex offenders commit sex offenses, the more likely they were to believe that juvenile sex offenders are unable to control their behavior and the less likely they were to support registration. Holding juvenile sex offenders less accountable for their actions and having less desire to punish them, in turn, predicted less registration support.

As expected, this pattern was obtained even though participants’ belief that childhood sexual abuse explains juveniles’ later sex offenses also predicted internal attributions to mental illness, deviant sexual arousal, and marginally greater estimations of recidivism. This is consistent with past research revealing that jurors sometimes perceive abuse as psychologically damaging (Stevenson et al., 2010; for review, see Stevenson, 2009). Even so, uncontrollable attributions and diminished retributive goals associated with attributing sex abuse to past abuse overshadowed the possible aggravating effects of negative internal attributions to mental illness and deviant sexual arousal. In other words, consistent with attribution theory (Weiner, 2006), we found that internal and stable attributions do not translate into unfavorable judgments when transgressions are also perceived as being caused by uncontrollable factors—in this case, a prior history of sexual abuse.

## Study 3

Study 3 tested whether Study 1 and 2 findings generalize to specific cases, because public support of sentencing policies tends to be stronger in the abstract as compared to when applied to specific cases in the context of support for the juvenile death penalty (Moon, Wright, Cullen, & Pealer, 2000); crime policy, punishment, and rehabilitation (Applegate, Cullen, & Fisher, 2002); three-strikes sentencing policies (Applegate, Cullen, Turner, & Sundt, 1996); and parental responsibility laws (Brank, Hays, & Weisz, 2006). In fact, Salerno, Najdowski, and colleagues (2010) found that people support sex offender registry laws for both adults and juveniles when they are asked about sex offenders in general, and that when asked to imagine a typical sex offender or offense, most people naturally envision sex offenders who commit the most heinous sex offenses such as rape and child sexual abuse. Yet, when given specific cases to consider, people supported registration less for younger juveniles and those who perpetrate less serious offenses (i.e., sexting, sexual harassment, statutory rape) as compared to older juveniles and those who perpetrate more serious offenses (i.e., forced rape). Therefore, in Study 3, we tested the extent to which beliefs that sexual abuse leads to sex offending influence registration support for juvenile sex offenders in specific cases (i.e., forced rape, statutory rape, harassment, and sexting). Because participants naturally envision severe prototypes of sex offenders when queried in the abstract, as in Studies 1 and 2 herein, we expected to replicate our findings that abuse attributions mitigate support for registration in a specific case involving a particularly severe offense (i.e., forced rape). Further, we anticipated that greater uncontrollable attributions and diminished retributive goals would mediate this mitigating effect, as in Study 2.

We had competing hypotheses regarding how beliefs linking sexual abuse to sex offending might influence registration support in less severe cases (i.e., statutory rape, harassment, and sexting). On the one hand, the mitigating effect already observed in Studies 1 and 2 and predicted for severe cases in Study 3 might generalize to less severe cases. On the other hand, such beliefs might be used in an aggravating way by increasing registration support in less severe cases, because participants might vary in the extent to which they perceive less severe acts to be developmentally normal sexual exploration rather than true crimes. In support, Salerno, Najdowski et al. (2010) found that even though the majority of participants (66%) did not support registration for less severe offenses, a significant minority of participants (34%) did. Further, it is possible that extralegal factors might alter the thresholds individuals have for determining whether certain sexual behaviors are labeled as normative versus deviant. For instance, Salerno et al. (2014) found that participants supported registration more for a less serious crime when the male juvenile offender was accused of having consensual sex with another underage boy than with an underage girl. Yet, this antigay bias did not emerge in the context of a serious crime involving an adult perpetrator and the underage victim. They theorized that when the crime is less serious, participants are more susceptible to expressions of bias that alter thresholds for labeling a sex act a crime. Stevenson et al. (2009) made a similar theoretical argument. In the context of a less serious crime (i.e., consensual oral sex), participants were more supportive of registering a juvenile when he and his victim were of different races than when they were of the same race. The authors theorized that, because interracial relationships are perceived as less normative and are generally less accepted, participants might have been more likely to label a less severe sex crime as a true crime when it was interracial rather than intraracial. Thus, it is possible that participants who think that juveniles commit sex offenses because they were sexually abused as a child might be more likely than others to interpret less severe sex acts between juveniles as true sex crimes. Further, we predicted that such an effect would be mediated by internal and stable attributions such that people who believe that a juvenile committed a sex offense because he was sexually abused might also believe that the juvenile is mentally ill, sexually deviant, and likely to commit future sex offenses. These attributions might, in turn, increase support for registration.

### Method

#### Participants

Participants were a diverse, urban group of undergraduates at a large Midwestern research university (*n* = 192) and community members (*n* = 83). Undergraduates were 56% women, 19 years old on average (*SD* = 1, ranging from 18 to 30 years), and 37% Caucasian, 32% Asian, 7% African American, 19% Hispanic, and 5% of other ethnicities. Community members were 56% women, 42 years old on average (*SD* = 17, ranging from 18 to 84 years), and 63% Caucasian, 8% Asian, 12% African American, 11% Hispanic, and 6% of other ethnicities. Again, the two samples were combined for analyses because analyses revealed no significant differences in their responses, all βs < .27, all *ns*.

#### Materials

Materials included the same questionnaire described in Study 2, modified by adding a second brief paragraph to describe a specific 16-year-old boy who had been found guilty of committing one of four specific sex offenses: (a) attacking and raping a girl in a park (forced rape), (b) participating in and videotaping mutually desired oral sex with an underaged girl (statutory rape), (c) running through school hallways grabbing girls’ buttocks (sexual harassment), or (d) getting caught looking at naked pictures of his underage girlfriend that she had e-mailed to him (sexting). The latter three offenses were based on actual cases in which sex offender registration was a possible outcome (*Wilson v. State of Georgia*, 2006; Goldsmith, 2007; and Stockinger, 2009; respectively). For example, in the sexting vignette, participants read:

While David (a 16-year-old male) was checking his e-mail in the school library, he received a message that contained naked pictures of his girlfriend. As he was viewing the pictures, a librarian walked by, noticed, and sent him to the principal’s office. After the school officials investigated, they discovered that the girl in the pictures was underage. David was adjudicated for possession of child pornography.

The other three vignettes were similar in length and level of detail.

Questionnaire items assessing (a) registration support; (b) beliefs that sexual abuse causes sex offending; (c) uncontrollable, internal, and stable attributions; and (d) utilitarian goals were the same as those described in Studies 1 and 2, except they were tailored to ask specifically about the juvenile described in the vignette (e.g., “Public registration laws are too severe for David’s case”). To assess the motivation underlying retributive goals of punishment more directly, we asked participants the extent to which they agreed that, “Listing David on the sex offender registry is an appropriate way to punish him for his offense.” Demographic items were the same.

#### Procedure

Seventy-nine percent of the undergraduates participated in the same type of mass-testing session as in Study 2, and 21% completed the questionnaire in a laboratory alone or in groups ranging from 2 to 24. Community members were recruited as in Study 2. Participants were randomly assigned to read about one of the four specific cases and then asked to complete all questions in response to the specific offense described in the vignette. When done, they were debriefed and thanked. Undergraduates were compensated with course credit for participating. Community members received no compensation.

### Results

Because our a priori definitions of the three less severe cases were that they would elicit less punitive case judgments than the fourth, more severe, case (as previous research has revealed, Salerno, Najdowski et al., 2010), we choose to simplify the presentation of our results by collapsing across the three less severe cases and presenting the results of our more severe case separately. Indeed, a planned contrast analysis comparing the case judgments of the fourth, severe case to the average case judgments of the three less severe cases revealed consistent significant effects such that participants endorsed more lenient (pro-offender) judgments in the less severe cases than the fourth, severe case (all βs > −.13, all *p* < .05) for all dependent variables except for internal attributions to deviant sexual arousal, β = .05, *ns*. Thus, our a priori definitions of the severity of the cases were largely supported by data analysis. Moreover, although we found a few significant differences between the three less severe case vignettes (i.e., statutory rape, harassment, and sexting) on some, but not all, dependent variables, *F*s(3, 261–268) > 8.11, *p*s < .01, these differences were uninteresting theoretically for the purposes of this research. Further, the range of mean differences between the three less severe cases on all dependent variables was much smaller (*M* range = .79) and was far outweighed by the range of mean differences between the average of the three less severe cases and the more severe case (i.e., forced rape) (*M* range = 1.81).

First, we present the main effects of abuse attributions on registration support, attributions, and punishment goals for the more severe case, followed by mediation analyses seeking to explain the main effect of abuse attributions on registration support. Then, we present the same analyses conducted for the less severe offenses.

#### More severe case

Analyses revealed that among participants who read about a juvenile who committed a more severe offense, those who expressed greater agreement that sexual abuse contributes to sex offending were significantly less supportive of the registry, β = −.46, *t*(38) = −3.17, *p* < .01, *R* = .46, *R*^2^ = .21, *F*(1, 38) = 10.09, *p* < .01; and made significantly more uncontrollable attributions, β = .31, *t*(39) = 2.02, *p* = .05, *R* = .31, *R*^2^ = .10, *F*(1, 39) = 4.09, *p* = .05; significantly more internal attributions to deviant sexual arousal, β = .37, *t*(41) = 2.56, *p* < .05, *R* = .37, *R*^2^ = .14, *F*(1, 41) = 6.56, *p* < .05; significantly fewer stable attributions, β = −.35, *t*(40) = −2.36, *p* < .05, *R* = .35, *R*^2^ = .12, *F*(1, 40) = 5.57, *p* < .05; and had significantly less retributive goals of punishment, β = −.38, *t*(41) = −2.64, *p* < .05, *R* = .38, *R*^2^ = .15, *F*(1, 41) = 6.95, *p* < .05. However, beliefs about sexual abuse did not have significant effects on internal attributions to mental illness, β = .23, *t*(40) = 1.50, *ns*, *R* = .23, *R*^2^ = .05, *F*(1, 40) = 2.25, *ns,* nor utilitarian goals, β = −.07, *t*(40) = −.47, *ns*, *R* = .07, *R*^2^ = .01, *F*(1, 40) = .223, *ns*.

Next, we conducted a series of regression analyses to determine which possible mediators explained the effects of beliefs that sexual abuse causes later offending on support for registering a juvenile who committed a more severe sex offense. We used the same mediation procedures described in Study 2. More stable attributions, β = .39, *t*(34) = 2.44, *p* < .05, and greater retributive goals, β = .39, *t*(34) = 2.44, *p* < .05, were associated with significantly greater registration support, but none of the other potential mediators emerged as significant predictors, all βs < −.15, *t*s < −.92, *ns*. Therefore, we tested whether stable attributions and retributive goals mediated the effect of beliefs that sexual abuse leads to sex offending on registration support for a juvenile convicted of a more severe offense.

Results from nonparametric bootstrapping analyses (as discussed above; see Preacher & Hayes, 2004; Preacher et al., 2007) based on 5,000 samples revealed no evidence of mediation. Specifically, the total effect of abuse attributions on registration support was significant (TE = −.65, *SE* = .21, *t* = −3.14, *p* < .05), yet the direct effect was also significant (DE = −.45, *SE* = .18, *t* = −2.49, *p* < .05). Indeed, neither stable attributions, 95% CI [−.16, .05] nor retributive goals, 95% CI [-.56,.02] significantly mediated the relationship between abuse attributions and registration support.

#### Less severe cases

Analyses considering only participants who read about juveniles who committed less severe offenses revealed that those who expressed greater agreement that sexual abuse contributes to sex offending were significantly more supportive of the registry, β = .26, *t*(228) = 4.10, *p* < .001, *R* = .26, *R*^2^ = .07, *F*(1, 228) = 16.84, *p* < .001; indicated significantly higher endorsement of retributive goals, β = .36, *t*(228) = 5.78, *p* < .001, *R* = .36, *R*^2^ = .13, *F*(1, 228) = 33.43, *p* < .001; made significantly more internal attributions to mental illness, β = .47, *t*(224) = 7.97, *p* < .001, *R* = .47, *R*^2^ = .22, *F*(1, 224) = 63.52, *p* < .001; significantly more internal attributions to deviant sexual arousal, β = .37, *t*(228) = 5.24, *p* < .001, *R* = .33, *R*^2^ = .11, *F*(1, 228) = 27.48, *p* < .001; significantly more stable attributions, β = .44, *t*(222) = 7.26, *p* < .001, *R* = .44, *R*^2^ = .19, *F*(1, 222) = 52.77, *p* < .001, and indicated significantly greater endorsement of utilitarian goals, β = .57, *t*(228) = 10.56, *p* < .001, *R* = .57, *R*^2^ = .33, *F*(1, 228) = 111.41, *p* < .001. Beliefs about sexual abuse did not significantly affect uncontrollable attributions, however, β = .03, *t*(226) = .41, *ns*, *R* = .03, *R*^2^ = .00, *F*(1, 226) = .17, *ns*.

**Figure 2 fig2:**
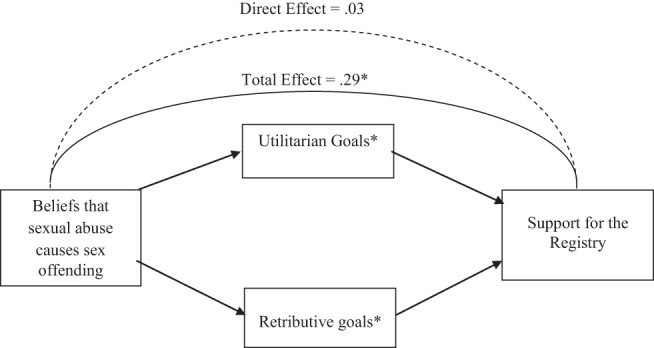
Mediators of the effect of beliefs that sexual abuse causes sex offending on support for the registry for the less severe sex offenses (Study 3). * *p* < .05.

Higher endorsement of retributive goals, β = .41, *t*(228) = 5.91, *p* < .001, and utilitarian goals, β = .42, *t*(227) = 6.99, *p* < .001, emerged as significant predictors of registration support, but none of the other potential mediators did, βs < .11, ts < −1.39, *ns*. Therefore, we tested whether retributive goals and utilitarian goals mediated the effect of beliefs about sexual abuse on support for registering juveniles convicted of less severe offenses (see Figure 2).[Fig-anchor fig2]

Supporting evidence of mediation, results based on 5,000 bootstrapped samples, revealed that the total effect of abuse attributions on registration support was significant (TE = .29, *SE* = .08, *t* = 3.95, *p* < .001), whereas the direct effect was not significant (DE = .03, *SE* = .08, *t* = .38, *ns*). Indeed, both retributive, 95% CI [.04, .20] and utilitarian goals of punishment, 95% CI [.05, .28] significantly mediated the effect of attributing a less severe sex offense to a history of being sexually abused on registration support (see Figure 2). Finally, a comparison of the relative strength of the individual indirect effects against each other revealed no significant difference, 95% CI [−.23, .13].

### Discussion

Results of Study 3 are consistent with past research showing that public support for sex offender registration varies depending on whether individuals are asked about juveniles in general or about specific juveniles accused of different crimes ranging in severity (Salerno, Najdowski et al., 2010; Salerno, Stevenson et al., 2010). The more participants thought that a juvenile’s history of being sexually abused led him to perpetrate forced rape, the less they supported registering the juvenile as a sex offender. These results, as well as Studies 1 and 2 and Salerno, Najdowski et al.’s (2010) results, support the idea that laypeople naturally think about heinous crimes when they are asked about sex offenders in general.

In contrast, the more participants thought that a history of sexual abuse led juveniles to perpetrate less severe offenses (i.e., statutory rape, harassment, and sexting), the more they supported registering the juvenile as a sex offender. Although some studies have shown that child sexual abuse mitigates reactions toward juveniles accused of nonsexual offenses (Najdowski et al., 2009; Nunez et al., 2007; Stalans & Henry, 1994), our results suggest that people sometimes use beliefs about a history of sexual abuse as an aggravating factor when determining whether juveniles should register as sex offenders for committing less severe sex offenses. Why? People sometimes consider abused offenders to be “damaged goods” (Najdowski et al., 2009; Stevenson et al., 2010). In fact, in less severe cases, beliefs linking sexual abuse to sex offending increased internal attributions to sexual deviance and mental illness, stable attributions in terms of perceived recidivism likelihood, retributive goals of punishment, and utilitarian goals of punishment.

Moreover, both retributive and utilitarian goals of punishment explained why beliefs about sexual abuse and sex offending increased support for registry laws, which is partially in line with attribution theory (Weiner, 2006) and our hypothesis that stable attributions triggering utilitarian goals would explain this aggravating effect. Some research indicates that although self-reported sentencing goals are utilitarian in nature (e.g., a desire to protect society; Ellsworth & Ross, 1983), actual sentencing goals primarily stem from a retributive desire to punish (Carlsmith, Darley, & Robinson, 2002; Darley, Carlsmith, & Robinson, 2000; Stevenson et al., 2010), perhaps explaining why both retributive goals and utilitarian goals mediated this effect. Furthermore, this finding is consistent with our hypothesis that participants who believe that a juvenile’s history of sexual abuse drove him to engage in sexual behavior might be more likely to interpret relatively less severe sex acts as true sex crimes and, in turn, treat the juvenile more punitively. In contrast, forced rape is probably always considered a true sex crime, regardless of beliefs about the causes of the perpetrator’s behavior (e.g., history of sexual abuse).

Finally, for the more severe offense, beliefs linking past sexual abuse to sex offending were associated with more uncontrollable attributions, more internal attributions to deviant sexual arousal, fewer stable attributions, and less retributive goals. These factors did not, however, mediate the effect of such beliefs on support for registering a juvenile who committed forced rape. This is in contrast to Study 2 findings, which indicated that uncontrollable attributions and diminished retributive goals explained that effect. Perhaps when participants are forced to consider an actual rape case, sympathy induced by the belief that the rapist was sexually abused may override other cognitions and attributions associated with this belief in a way that does not happen when participants are asked to consider a sex crime in the abstract. In support, much research shows that participants tend to be more sympathetic toward offenders when considering specific perpetrators of crime rather than criminal acts in general (e.g., Applegate et al., 2002; Brank et al., 2006; Moon et al., 2000).

## Study 4

Thus far, we provided indirect evidence that the belief that sexual abuse leads to sex offending reduces support for registering juveniles who commit serious sex offenses, whereas this belief increases support for registering juveniles who commit less severe sex offenses. To conclude this with more certainty about causality, we conducted a direct experimental test to understand how a juvenile’s history of sexual abuse influences registration support in a forced rape case (Study 4) and separately in a statutory rape case (Study 5). Again, this work was guided by attribution theory and tested competing hypotheses. On the one hand, a history of being sexually abused as a child (relative to none) might cause participants to believe that the juvenile was less able to control his sexual behavior, which should, in line with Graham and colleagues’ (1997) results, diminish retributive goals and reduce registration support. On the other hand, knowing that a juvenile sex offender was sexually abused as a child might predict greater internal attributions for juveniles’ sex offending to factors such as deviance or mental illness, as well as stable attributions that the juvenile is likely to reoffend and, in turn, greater registration support.

Yet in Study 3, abuse attributions predicted diminished registration support in the severe rape case but greater retributive and utilitarian goals and greater registration support in the lenient cases. We theorized that participants were more likely to interpret the relatively less severe sex crimes (e.g., statutory rape) as more like true sex crimes when the juvenile had been sexually abused himself as a child. In contrast, believing the juvenile had a history of sexual abuse might not have influenced perceptions of whether forced rape is a true sex crime because participants probably perceived forced rape as an unambiguous sex offense. Thus, in Study 4, we tested the extent to which this pattern of results would generalize when we experimentally manipulated abuse history in the context of a more severe sex crime—forced rape.

### Method

Study 4 conformed to a one-way between-subjects experimental design in which the sexual abuse history (abused or nonabused) of a juvenile who committed forced rape was experimentally manipulated.

#### Participants

Participants were 82 community members chosen to be nationally representative. Forty-four participants (54%) were in the abused condition and 38 (46%) were in the nonabused condition. Ten participants failed the manipulation check (i.e., three participants in the abused condition said the juvenile was not abused and seven participants in the nonabused condition said the juvenile was abused). These participants were excluded from analyses, resulting in a final sample size of 72, which was 54% women, 39 years old on average (*SD* = 9, ranging from 20 to 62 years), and 73% Caucasian, 6% Asian, 11% African American, 7% Hispanic, and 2% of other ethnicities.

#### Materials

Materials included the same description of the forced rape case followed by the same questions used in Study 3, with these exceptions: To accommodate the sexual abuse manipulation, we described the juvenile as having been “sexually abused by his father when he was a child” or as having “no history of being sexually abused as a child.” We did not measure participants’ beliefs that sexual abuse causes sex offending because we manipulated abuse history. We included an additional measure of support for the full application of the registry, developed from an item used by Salerno, Najdowski et al. (2010) and Stevenson et al. (2009). Specifically, we asked participants, “In your opinion, what is the most appropriate outcome for David?” Response options were 1 (*should not be required to register*), 2 (*should be required to register, but his information should not be posted on the Internet*), 3 (*should be required to register, but his information should not be posted on the Internet until he turns 18, at which time his information should be publicly posted on the Internet*), and 4 (*should be required to register and his information should be publicly posted on the Internet*), with higher numbers indicating greater support for the full application of the registry. Finally, a manipulation check item asked participants to respond (yes or no) to the question, “Was the juvenile offender sexually abused as a child?”

#### Procedure

Community members were recruited via StudyResponse, a nationally representative database from which participants are recruited and given a $5 incentive to participate. Participants completed the questionnaire online, were thanked, and given their incentive, in keeping with IRB-approved procedures.

### Results and Discussion

We conducted a series of one-way analyses of variance (ANOVAs) to test the effect of abuse history (abused or nonabused) on participants’ registration support; degree of support for the full application of the registry; internal, uncontrollable, and stable attributions for offending; and retributive goals of punishment. There were no significant effects of abuse history on any case judgments, all *F*s(1, 57–70) ≤ 2.66, *ns*. Thus, although Studies 1, 2, and 3 showed that participants’ abuse attributions predict lenient treatment of juvenile sex offenders, in a true experimental test of the influence of abuse history, participants did not use abuse history as a mitigating factor. Instead, participants appeared to ignore abuse history, just as they frequently discount adult defendants’ histories of child physical abuse as a mitigating factor in death penalty cases (Stevenson et al., 2010).

## Study 5

In Study 5 we experimentally manipulated abuse history in the context of a less severe sex crime, statutory rape, to replicate and extend Study 4 to a type of sex crime with which juveniles are more commonly charged.

### Method

Study 5 conformed to a one-way between-subjects experimental design in which the sexual abuse history (abused or nonabused) of a juvenile who committed statutory rape was experimentally manipulated.

#### Participants

Participants were 78 community members chosen to be nationally representative. Thirty-nine participants (50%) were in the abused condition and 39 (50%) were in the nonabused condition. Eight participants failed the manipulation check (i.e., two participants in the abused condition said the juvenile was not abused and six participants in the nonabused condition said the juvenile was abused). These participants were excluded from analyses, resulting in a final sample size of 70, which was 50% women, 38 years old on average (*SD* = 9, ranging from 22 to 65 years), and 68% Caucasian, 7% Asian, 12% African American, 12% Hispanic, and 3% of other ethnicities.

#### Materials and procedure

Materials included the same descriptions of the less severe cases (i.e., statutory rape, harassment, and sexting) as in Study 3, with all the same questions and procedures used in Study 4.

### Results

We conducted the same series of ANOVAs as in Study 4 to test the effect of abuse history on participants’ case judgments.

Participants were marginally more supportive of the full application of the registry for the abused (*M* = 2.30, *SD* = 1.29) than the nonabused juvenile (*M* = 1.75, *SD* = 1.05), *F*(1, 67) = 3.67, *p* = .06. Participants rated the abused juvenile as significantly more likely to be mentally ill (*M* = 3.06, *SD* = 3.01), significantly more likely to commit future sex crimes (*M* = 4.19, *SD* = 2.60), and marginally less able to control his behavior (*M* = 2.62, *SD* = 1.09) than the nonabused juvenile (*M* = 1.31, *SD* = 2.26; *M* = 2.47, *SD* = 2.59; and *M* = 2.12, *SD* = 1.10, respectively), *F*(1, 66) = 7.13, *p* = .01; *F*(1, 67) = 7.53, *p* < .01; and *F*(1, 67) = 3.54, *p* = .06, respectively. Participants also endorsed marginally greater retributive and utilitarian goals of punishment when the juvenile was abused (*M* = 2.81, *SD* = 1.31; and *M* = 2.47, *SD* = 1.13, respectively) than not abused (*M* = 2.25, *SD* = 1.32; and *M* = 1.94, *SD* = 1.16, respectively), *F*(1, 67) = 3.13, *p* = .08; and *F*(1, 67) = 3.68, *p* = .06, respectively. There was no main effect of abuse history on registration support nor attributions to deviant sexual arousal, all *F*s(1, 43–67) < 1.36, *ns.*

**Figure 3 fig3:**
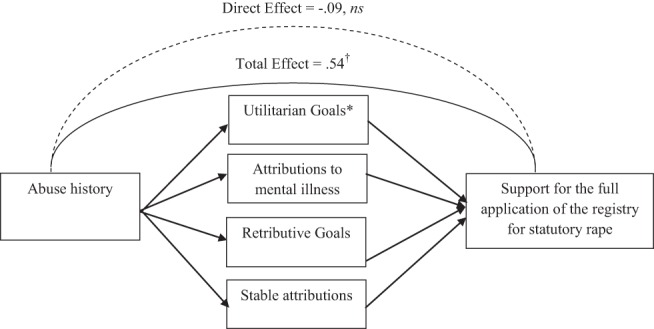
Mediators of the effect of abuse history on support for the full application of the registry for the statutory rape sex offense (Study 5). ^†^ *p* < .10, * *p* < .05.

Next, although the effect of abuse history on support for the full application of the registry was only marginally significant, it was in line with our theoretically driven hypotheses, and so we conducted analyses to explore possible mediators of that effect (see Figure 3). Because ANOVAs revealed no relationship between abuse history and attributions to deviant sexual arousal, according to Baron and Kenny (1986), this variable could possibly mediate the relationship between abuse history and support for the full application of the registry, and was therefore not considered for mediation analyses. Stable attributions emerged as a statistically significant predictor of support for the full application of the registry, β = .62, *t*(64) = 5.70, *p* < .001, as did uncontrollable attributions, β = −.20, *t*(64) = −2.13, *p* < .05, retributive goals, β = .68, *t*(67) = 7.58, *p* < .001, and utilitarian goals, β = .72, *t*(66) = 8.32, *p* < .001. Attributions to mental illness emerged as a marginally significant predictor of support for the full application of the registry, β = .20, *t*(64) = 1.82, *p* = .07.[Fig-anchor fig3]

Although the effect of uncontrollable attributions on support for the full application of the registry was significant, this effect was in the opposite direction as the effect of abuse history on support for the full application of the registry. That is, although participants were more supportive of the full application of the registry for the abused juvenile than the nonabused juvenile (i.e., a punitive judgment), the abused juvenile was also perceived as less able to control his behavior—a variable that predicts less support for the full application of the registry (i.e., a lenient judgment). Because uncontrollable attributions are lenient (prodefense) judgments, and the abused juvenile was rated as less able to control his behavior, this belief logically cannot explain why participants were more punitive toward the abused than nonabused juvenile (i.e., more supportive of the full application of the registry), and so this variable will no longer be considered.

We were, however, able to test whether stable attributions, attributions to mental illness, retributive goals, and utilitarian goals mediated the effect of abuse history on support for the full application of the registry (see Figure 3). Supporting evidence of mediation, results based on 5,000 bootstrapped samples, revealed that the total effect of abuse attributions on registration support was marginally significant (TE = .54, *SE* = .29, *t* = 1.87, *p* = .07), but the direct effect was not significant (DE = −.09, *SE* = .19, *t* = −.49, *ns*). Yet, only utilitarian goals emerged as a significant mediator, 95% CI [.02, .58]. Retributive goals, attributions to mental illness, and stable attributions did not emerge as significant mediators, 95% CIs [−.05–.00, .40–.52] (see Figure 3). Finally, a comparison of the relative strength of the individual indirect effects against each other revealed no significant differences, 95% CIs [−.51–.13, .27–.53]. Thus, participants supported the full application of the registry more for the abused than the nonabused juvenile because they were more likely to believe he posed a danger to society.

### Discussion

Although abuse history did not influence the registration support variable, participants were marginally more supportive of the full application of the registry for a sexually abused juvenile than a nonabused juvenile who had committed statutory rape. Also, an abused juvenile who committed statutory rape was perceived as more mentally ill, less able to control his behavior, and more likely to recidivate than a nonabused juvenile. Participants also endorsed marginally greater retributive and utilitarian goals for the abused than the nonabused juvenile. Further, mediation analyses revealed that utilitarian goals of punishment (i.e., the belief the juvenile was a danger to society) drove the effect of abuse history on support for the full application of the registry. Interestingly, participants were more supportive of registering an abused versus a nonabused juvenile who committed statutory rape, even though they believed the abused juvenile was less able to control his behavior—an attribution that both the present and past research (Weiner, 2006) shows predicts leniency in case judgments. The present research demonstrates an interesting instance in which fear of recidivism (i.e., utilitarian goals of punishment) overrides the leniency that would otherwise be produced by uncontrollable attributions, and instead results in severe case judgments. Thus, just as in Study 3, participants might be more likely to label a relatively less severe sex crime (i.e., statutory rape) as a true crime when the juvenile has a history of sexual abuse and, in turn, use his history of abuse as evidence that he is permanently damaged, a danger to society, and deserving of registration. This is in line with past work illustrating that participants sometimes use abuse history as an aggravating factor (Najdowski et al., 2009; Stevenson et al., 2009).

It is noteworthy that abuse history predicted support for the full application of the registry, but not registration support. It is possible that the registration support variable triggers retributive goals of punishment because its wording refers to the severity of registration (i.e., “Public registration laws are too severe for juvenile sex offenders like David”). In contrast, the question assessing support for the full application of the registry does not require participants to consider the punitive severity of registration. Instead, participants are merely asked to recommend one of various registration options (i.e., no registration; registration, but without the juvenile’s information posted online; etc.) without making a value judgment about those options. In support, the effect of abuse history on support for the full application of the registry was not mediated by retributive goals of punishment but instead utilitarian goals to protect society.

## General Discussion

As expected, when asked about juvenile sex offenders generally, participants greatly overestimated the prevalence of a history of sexual abuse among juvenile sex offenders, just as they overestimate histories of sexual abuse for adult sex offenders (Fortney et al., 2007; Levenson et al., 2007). In line with attribution theory (e.g., Weiner, 2006), when asked in the abstract, the more participants attributed sex offending to past abuse, the less they supported policies that require juveniles to register as sex offenders. Further supporting attribution theory, this effect was significantly mediated by uncontrollable attributions and retributive goals of punishment. These results are in line with Stevenson and colleagues’ (2010) research, which showed that uncontrollable attributions about a defendant’s history of having been physically abused as a child predicted lenient sentence preferences (i.e., life over death).

Yet when participants were asked to consider specific cases, attributions to abuse reduced support for juvenile registration policies only for severe sex crimes like rape, which, unsurprisingly, are the very types of crimes that participants naturally tend to envision when asked generally about sex crimes (Salerno, Najdowski, et al., 2010). For less severe juvenile sex crimes, however, the more participants attributed sex offending to past abuse, the more they supported registration. Finally, when a history of sexual abuse was experimentally manipulated, abuse history was consistently used as an aggravating factor in a less severe statutory rape case, consistent with what Najdowski et al. (2009) found for a juvenile who committed crimes against someone other than the perpetrator of abuse, and was ignored entirely in a severe forced rape case.

Although the results of these studies might seem inconsistent, they are in line with existing theory and our hypotheses. That is, serious types of sexual crimes are considered prototypical sex crimes (Salerno, Najdowski et al., 2010), and in turn, participants’ use of abuse history mirrors how it is used when asked about sex crimes generally: Abuse history mitigates support for juvenile sex offender registration. Yet, for nonprototypical (but more common; U.S. Department of Justice, 2007) less serious sex crimes, a different trend emerges, in large part due to the malleability of the perceived seriousness of the sexual offense—malleability that does not exist for extremely serious types of sexual offenses. Specifically, less severe sex crimes (i.e., statutory rape) are more likely to be perceived as true crimes when the juvenile has a history of sexual abuse and, in turn, participants use a juvenile’s sexual abuse history as evidence that he is permanently damaged, a danger to society, and deserving of registration.

### Public Policy Implications

Throughout this manuscript, we have discussed the social psychological implications of our work in terms of attribution theory and decision making. Our results, however, also have a number of implications that inform child-related public policy and law (Stevenson et al., 2009; for a review, see Bottoms, Najdowski, & Goodman, 2009). Most people inaccurately assume juvenile sex offenders have been abused. Moreover, normative and consensual adolescent sexual activity is particularly criminalized when people assume that the adolescent is engaging in sexual activity because of his own history of abuse. Such findings have implications with respect to the fairness of registration policies, particularly because most juvenile sex offenders have not been sexually abused (Ryan et al., 1996). To the extent that judges are allowed judicial discretion in applying registration policies to adolescents, the present research suggests that juvenile registration is likely to be applied capriciously and affect certain groups more than others.

This research also has implications for sentencing. Sexual abuse history, presumed by the law to be a mitigating factor (e.g., Illinois’ Juvenile Court Act, 1987), is at best frequently discounted, especially in severe cases, and at worst, even backfires in lenient cases, being used against juvenile sex offenders as an aggravating factor, as are other factors such as drug abuse, alcohol abuse, and child physical abuse (Barnett, Brodsky, & Price, 2007; Brodsky, Adams, & Tupling, 2007; Najdowski et al., 2009; Stevenson et al., 2010; for review, see Stevenson, 2009). This is especially noteworthy considering that less severe sex crimes constitute the majority of juvenile sex offenses (U.S. Department of Justice, 2007). This finding is likely to be of interest to trial attorneys who must attempt to anticipate the factors that jurors will consider aggravating versus mitigating.

Although laws such as the Illinois Juvenile Court Act (1987) mandate that child abuse be considered a mitigating factor, evidence suggests that the opposite is happening for child physical abuse (Stevenson, 2009) and, in some cases, child sexual abuse (e.g., Najdowski et al., 2009). Yet, because the current research demonstrates that abuse attributions mitigate case judgments when participants consider juvenile sex offenses in the abstract, it is likely that legal decision makers and the general public assume that child abuse is being used as a mitigating factor. These assumptions undermine the effectiveness of such laws. Courts and policymakers should be encouraged to implement legal instructions and policies designed to encourage legal decision makers explicitly to be sensitive to a juvenile offender’s history of abuse, to educate them about the actual consequences of being abused, and to admonish them against using a history of abuse against a juvenile offender. It may be prudent for legal decision makers to provide rehabilitative resources and mental health services to juvenile sex offenders who commit these less severe nonviolent offenses, particularly those with histories of child sexual abuse, instead of resorting to potentially harmful sex offender registration (Salerno, Stevenson et al., 2010).

Given that participants greatly overestimate the prevalence of a history of sexual abuse among juvenile sex offenders, and that this belief can lead to more severe treatment of juvenile sex offenders, another policy implication is to educate legal decision makers about actual prevalence rates of abuse histories among juvenile sex offenders. Although only a small minority of sexually abused individuals become sex offenders (Worling, 2001), due to well-documented human reliance on heuristics in decision making (e.g., Kahneman & Tversky, 2000), these common errors in thinking and illusory correlations are likely to continue, particularly if not corrected. Policy-focused educators should take precautions when teaching this information and be careful to correct such mistakes in logic—mistakes that have the potential to result in discriminatory treatment of sexually abused juveniles.

### Limitations and Future Directions

Abuse history influences the types of attributions laypeople made about a juvenile’s sex offending and their goals for sentencing a juvenile, but these attributions and goals did not consistently explain the effects abuse had on laypeople’s support for registering a juvenile as a sex offender. Perhaps this is explained by the fact that internal attributions to sexual deviance and mental illness could be perceived as either controllable or uncontrollable as well as either permanent or transitory. In fact, because of the potential for confounds among the dimensions of controllability, locus, and stability, Weiner (1985, 2006) has argued that attributions of controllability are most central to understanding people’s beliefs about the causes of behavior. Even so, uncontrollable attributions significantly explained the effects of a juvenile’s past history of abuse on public support for sex offender registration in only one of our studies. Future research might better test whether participants use abuse as a mitigating or aggravating factor by teasing these confounds apart; for example, by experimentally manipulating whether internal attributions for sexual deviance or mental illness are viewed as either stable or unstable and controllable or uncontrollable. Also, future research could provide a more complete test of Weiner’s (2006) attribution theory by exploring whether affective reactions, such as sympathy and anger, mediate the effects of abuse attributions and abuse history on perceptions of juvenile sex offenses. Future studies should also explore how abuse attributions influence policymakers’, juvenile justice officials’, and other legal decision makers’ perceptions of juvenile sex offenders.

Our methods were realistic in several regards: the vignettes were modeled after real cases, and we employed both undergraduate and representative community member samples in an attempt to obtain generalizable results. A key methodological finding, aside from all our central findings related to our theories about reactions to juvenile offenders, is that there were no differences between these two types of samples. The fungibility of undergraduate and community jurors has long been debated in the field of psychology and law. In understanding reactions to juvenile offenders, our work stands in support of research with undergraduate subjects as a proxy for community members.

Although the purpose of this research was to test factors that shape public support for social policy, future research should test the possibility that these results generalize to a trial context. Indeed, replication of this line of research in mock trial contexts, with more realism and ecological validity, including lengthier trial transcripts and jury deliberations, is prudent. For instance, participant samples were primarily White, and future research should include more diverse participant samples. In addition, participants were provided with minimal information explaining sex offender registration policy and the sexual crime vignettes were short and did not contain a great deal of case-related evidence. Even so, no psycho-legal study will fully replicate real events, nor is complete replication necessary to test basic psychological mechanisms underlying some decisions (e.g., Golding, Dunlap, & Hodell, 2009). Our work is a necessary first step in understanding how abuse attributions and abuse history influence public support for policies affecting juvenile sex offenders.

## Conclusion

Examining how a juvenile sex offender’s history of sexual abuse shapes support for registration policies across a series of five studies with various methodologies and case types certainly gets us closer to a fuller understanding of public attitudes toward particularly vulnerable and young offenders. The questions addressed by this research are critical given that registering juveniles is not only ineffective at reducing sex offenses, but also potentially negatively influences the lives of those registered in ways that could contribute to future recidivism (see, e.g., Levenson et al., 2007). Understanding biases against juvenile offenders who have already experienced maltreatment (i.e., sexual abuse) is one important step toward the development of future policy designed to combat discrimination against victimized and vulnerable young offenders.
